# Reconstruction of a genome-scale metabolic model for *Streptococcus zooepidemicus*: Comparison with *Corynebacterium glutamicum* to study hyaluronic acid production

**DOI:** 10.1371/journal.pone.0335509

**Published:** 2025-12-31

**Authors:** Zahra Nikuiyan, Fatemeh Tabandeh, Ehsan Motamedian, Saeed Salehi, Marjan Talebi, Rouzbeh Almasi Ghale

**Affiliations:** 1 Department of Energy and Environmental Biotechnology, National Institute of Genetic Engineering and Biotechnology (NIGEB), Tehran, Iran; 2 Department of Biotechnology, Faculty of Chemical Engineering, Tarbiat Modares University, Tehran, Iran; 3 Department of Pharmacognosy, TeMS.C., Islamic Azad University, Tehran, Iran; 4 Herbal Pharmacology Research Center, TeMS.C., Islamic Azad University, Tehran, Iran; Universitat fur Bodenkultur Wien, AUSTRIA

## Abstract

Comparison of *Streptococcus zooepidemicus* (*S. zooepidemicus*) as a natural strain for hyaluronic acid (HA) production with *Corynebacterium glutamicum* (*C. glutamicum*) as a recombinant host is a valuable tool to identify key metabolic targets for enhanced HA production. For this purpose, a comparative analysis based on the genome-scale metabolic networks of native and recombinant HA producers was conducted. In this study, the first genome-scale metabolic model (GEM) of *S. zooepidemicus*, was reconstructed and named i*ZN522*. This systematically curated model was validated using various biochemical data, consisting of growth rate prediction, amino acids auxotrophy, and different carbon sources consumption. Following the validation, key genes for HA overproduction in both bacteria were identified through iterative single-gene deletion analysis. Comparing optimal flux distributions predicted by models of *S. zooepidemicus* strain with recombinant *C. glutamicum* revealed that removing the oxidative pentose-phosphate pathway in *C. glutamicum* effectively increases the hyaluronan synthase flux. Additionally, improvement strategies in *C. glutamicum* primarily rely on up- and down-regulation, while in *S. zooepidemicus*, gene deletions can optimize the HA production rate. By comparing the two metabolic models, it can be inferred that eliminating of glucose-6-phosphate dehydrogenase enzymatic reaction in *C. glutamicum* results in greater similarity in the central carbon metabolisms of *C. glutamicum* and *S. zooepidemicus*, thereby enhancing HA production.

## 1. Introduction

A genome-scale metabolic model (GEM) contains curated gene-protein-reaction (GPR) associations based on annotated genome and experimental observations. These models provide a quantitative format to predict the phenotypes of the target organism in a specific steady-state condition [1–3]. GEMs are becoming increasingly powerful tools for understanding and manipulating microbial metabolism and enable in-depth understanding of biological big data, with recent reviews highlighting their applications in investigating microbial metabolic adaptations [4,5]. Moreover, GEMs allow us to identify optimized flux distributions with computational tools such as flux balance analysis (FBA), which utilize linear programming solvers [3].

Hyaluronic acid is a non-immunogenic linear glycosaminoglycan composed of repeating disaccharide units of D-glucuronic acid and N-acetyl-D-glucosamine [6–8]. Due to its unique physicochemical properties, including high viscosity, biocompatibility, and hygroscopicity, HA is a biomaterial with a broad range of applications in various fields, including pharmaceuticals, cosmetics, biomedicine and the food industry [9–12]. The global HA market size was valued at United States dollar (USD) 8.3 billion in 2018 and is anticipated to reach USD 15.25 billion in 2026 [10]. Indeed, its expanding use across diverse sectors including ophthalmology, rheumatology for joint lubrication, advanced wound care, and tissue engineering, alongside significant demand from the pharmaceutical industry for drug delivery systems and the cosmetics sector for sophisticated skincare formulations, underpins this market growth [12,13]. Consequently, efficient and sustainable biotechnological production routes are increasingly critical to meet these varied and growing demands [7,12,14]. Recent market analyses affirm this trajectory, indicating sustained high demand and significant economic value, further motivating research into optimized bioproduction routes [15]. In recent years, to meet the market demands and circumvent issues with animal-derived HA, such as potential viral contamination and ethical concerns, HA is mainly provided by microbial fermentation [7,8]. Native and recombinant bacterial systems are used for HA production [16]. *Streptococcus equi* subsp. *zooepidemicus* strains are commonly used as native HA producers, capable of producing 6–7 g/L HA, under appropriate culture conditions [6,17,18]. However, as a biosafety level 2 opportunistic pathogen, its industrial use necessitates stringent safety and purification protocols [18]. Recently, heterologous HA biosynthesis by GRAS (generally recognized as safe) recombinant producers such as *Corynebacterium glutamicum* has come into consideration as a promising alternative [19–22]. While significant progress has been made in engineering these GRAS hosts, HA yield produced by recombinant *C. glutamicum* (as a recombinant HA producer) is lower than the HA produced by S. *zooepidemicus* (as a native HA producer), presenting ongoing challenges for metabolic engineering [16,20,23]. The HA capsule, a virulence factor for the animal pathogenic bacterium *S. zooepidemicus,* plays a vital role in evading the host immune system [18]. It seems that the metabolic pathways of *S. zooepidemicus* are adopted for HA biosynthesis in an efficient way. *S. zooepidemicus* uses approximately 5% of the carbon source for HA biosynthesis, but 10% and 80% of carbon sources were consumed for biomass generation and lactic acid production, respectively [24]; thus, growth rate competes with HA production for carbon source and energy [25], and experimental observations has confirmed this statement [24,26]. Many attempts have focused on improving the fermentation conditions [17,27–29] and medium composition [30–32] to overproduce HA in *S. zooepidemicus*. Overexpression of NADH oxidase as a metabolic engineering approach in *S. zooepidemicus* led to a 15% and 33% increase of biomass and ATP, respectively. Nevertheless, the HA yield remained constant, often because other factors like precursor availability or synthase activity subsequently become limiting [8,18]. Experimental observations have shown that redirecting carbon flux from glycolysis to the HA synthesis pathway improves HA productivity. For instance, the strategy of expressing polyhydroxybutyrate (PHB) synthesis genes (phbCAB) in *S. zooepidemicus* has been investigated as a means to create an alternative carbon sink. This approach, particularly when combined with the inhibition of lactic acid synthesis, reportedly increased the HA production from 5.5 g/L to 7.5 g/L [8,18]. Recombinant *C. glutamicum* produces lactic acid as the main by-product in the HA fermentation process. Cheng et al. (2017) [33] have reported that knockout of the lactate dehydrogenase gene in recombinant *C. glutamicum* increased HA titer significantly. The evaluation of *C. glutamicum* GEM, named i*CW773*, as detailed by Cheng et al. (2019) [20], indicated that glycolysis pathway attenuation, PPP knockout, lactate/acetate pathway deletion, and PDH activity attenuation enhanced HA titer to 27.8 g/L in fed-batch culture.

While extensive research has been conducted to optimize HA production in *S. zooepidemicus* and recombinant *C. glutamicum* [18,21] including recent efforts to control HA molecular weight through precursor flux engineering in *S. zooepidemicus* [34], no studies have investigated the optimization of the whole-cell metabolic networks of *S. zooepidemicus* for HA production using a newly reconstructed and comprehensively validated GEM for this specific purpose. In addition, there is no report comparing these bacteria via detailed computational systems biology and comparative GEM analysis to identify target genes for increasing HA production rate. Because of the HA pathway’s complexity and its relationship with biomass and ATP, along with the recognized influence of transcriptional regulatory networks on HA synthesis [35], a comprehensive metabolic model can help answer these questions. Such models, especially when standardized [36] and built upon current genomic and biochemical knowledge [37,38], provide a robust platform for *in silico* exploration.

In this study, a GEM was employed to explore novel strategies for enhancing the HA production rate. To accomplish this goal, the GEM of *S. zooepidemicus* ATCC 35246 was reconstructed for the first time and denoted as i*ZN522*. Subsequently, the differences in optimal flux distributions between the native and recombinant producers were analyzed by the FBA method. Moreover, single- and multiple- gene knockout for HA optimization in both strains were systematically identified by FBA and iterative minimization of metabolic adjustment (MOMA) methods. Additionally, recombinant *C. glutamicum* GEM was evaluated computationally and strategies were introduced for HA production rate enhancement. This work aims to bridge the gap in comparative systems-level understanding of HA production in these key microorganisms.

## 2. Materials and methods

### 2.1. Genome-scale metabolic model reconstruction

The *S. zooepidemicus* GEM was reconstructed based on genome annotation data and experimental data from the literature according to the procedure presented by Thiele and Palsson [39]. While also incorporating best practices and considerations for model quality outlined in recent GEM reconstruction guidelines [5,38,40]. The reconstruction of the model was carried out in three steps: generation of the draft reconstruction, manual refinement of the draft reconstruction, and determining the pseudo reactions of biomass and HA.

The first step consisted of collecting and organizing the metabolic reactions of *S. zooepidemicus*, including specific pathways for HA biosynthesis, using biochemical data for the ATCC 35246 strain, based on its genome sequence (GenBank access code: CP002904.1) published by Ma et al. [41] provided by the KEGG database [42], transport reactions listed in transportDB [43], and corresponding metabolites for each reaction elicited from PubChem [44] and ChEBI [45] in an EXCEL file. Automated tools like CarveMe can assist in generating initial draft reconstructions from annotated genomes, which then require extensive curation [46]. For manual curation in the second step, initially, the names, formation, and direction of reactions were checked using the BIGG database [47]. Then, using Marvin View software 14.7.7, 2014, developed by ChemAxon (chemaxon.com), the metabolite’s charges were estimated and following that, the reactions were charge-balanced. Finally, the biochemical literature of *S. zooepidemicus* was reviewed, and new reactions were added to the draft file.

In the third step, experimental data for biomass composition were not available for *S. zooepidemicus*, and hence, the molar ratios for deoxyribonucleotides of DNA and ribonucleotides of RNA were calculated using the genome and the whole-cell RNA sequence of *S. zooepidemicus* provided by (microbedb.jp) and (bacteria.ensembl.org). Protein composition was calculated using codon preference data of *S. zooepidemicus* (dnahive.fda.gov). A detailed calculation of DNA, RNA, and protein composition is presented in the Supplementary File S1 in S1 File. Other data for the biomass reaction were obtained from the closest strain, *Lactococcus lactis* [48]. Formulating an accurate biomass objective function is crucial for GEM predictions, and guidelines for its systematic construction are available [37–39].

**Fig 1:**
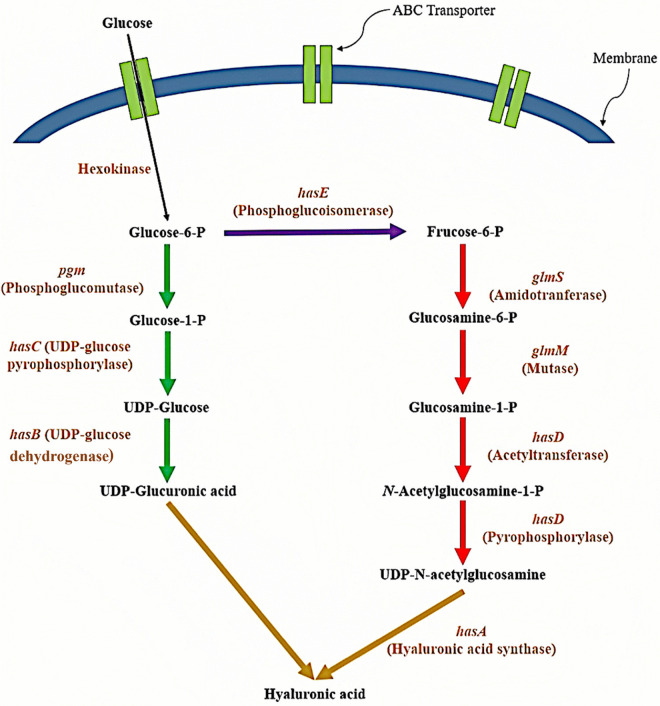
Hyaluronic acid biosynthesis pathway in *S. zooepidemicus.* This schematic illustrates the biosynthetic pathway of hyaluronic acid (HA) emphasizing the metabolic precursors and enzymatic steps. The pathway starts from glucose, which is metabolized to form two activated sugar precursors: UDP-glucuronic acid (UDP-GA) and UDP-N-acetylglucosamine (UDP-GlcNAc). These precursors are polymerized by the enzyme hyaluronan synthase, encoded by the *hasA* gene, to form HA. Arrows indicate the direction of enzymatic reactions. Key energy inputs and cofactors are labeled. This figure contextualizes the metabolic investment required for HA synthesis, highlighting the critical nodes for regulation and genetic manipulation. Abbreviations: ATP, adenosine triphosphate; NAD, nicotinamide adenine dinucleotide; UTP, uridine triphosphate.

Fig 1 represents the HA biosynthetic pathway in *S. zooepidemicus*, showing two precursors consisting of UDP-glucuronic acid (UDP-GA) and UDP-acetylglucosamine (UDP-GlcNac) linked by a polymerase called hyaluronan synthase (encoded by *hasA*) [49,50]. The biosynthesis of the HA disaccharide unit consumes five ATP molecules, one acetyl-CoA, and two NAD cofactors [8,18]. The pseudo reaction of the HA formation and the HA exchange reaction were added to the draft file to simulate HA production by this model. The growth-associated maintenance (GAM) energy described as the amount of ATP consumed for biomass formation, was taken from the *L. lactis* model (39.4 mmol ATP.gDCW^-1^.h^-1^) [48]. Non-growth associated maintenance (NGAM) energy was calculated based on the experimental glucose consumption rate presented by Blank et al. and Hamilton et al. [51,52]. For more detailed NGAM calculation, see the Supplementary File S1 in S1 File.

### 2.2. Growth simulation and validation

Model simulations were carried out using the Systems Biology Markup Language (SBML) Level 3 [53], Constraint-Based Reconstruction and Analysis (COBRA) toolbox version 3.0 [54], and the GNU Linear Programming Kit (GLPK) package in MATLAB software. FBA [55] was applied to predict optimal flux distributions in different media and conditions. The intracellular reversible reaction fluxes were limited to between −1000–1000 mmol.gDCW^-1^.h^-1^, and the intracellular irreversible reaction fluxes were set between 0 and 1000 mmol.gDCW^-1^.h^-1^. The lower and upper bounds of other exchange reactions were calculated using Eq. 1, based on previous experimental data [51]. The glucose uptake rate was set to 18.56 mmol.gDCW^-1^.h^-1^ in all simulated conditions, and the exchange reaction of oxygen was set to zero to simulate anaerobic conditions. Other constraints of exchange reactions are presented in S1 Table in S1 File.


$${\text{q}}_{\text{i}}=\text{D}.\frac{Ci.supernatant-Ci.feed}{Xbiomass}$$ (1)

The q_i_ is the flux of the corresponding metabolite in mmol.gDCW^-1^.h^-1^, Ci presents concentration of metabolite i in mmol per liter, Xbiomass is the biomass concentration in gDCW/L, and D is the dilution rate (h^-1^).

For the growth simulation, two chemically defined media named CDM and CDM1, and a complex medium were used. The compositions of these culture media are provided in **S2** and **S3 Tables** in S1 File. For the growth prediction on complex media including yeast extract, the chemical composition was estimated according to the data presented by Oh et al. [56]. The objective function was set to biomass reaction to simulate the growth of *S. zooepidemicus* in all *in silico* experiments.

The growth rate prediction using a model is a crucial stage for model validation. Standardized model testing suites like MEMOTE [36] offer comprehensive quality control checks beyond basic growth prediction. To evaluate the predicted growth rate by our model, growth simulation on CDM medium in anaerobic conditions was performed using FBA based on the experimental data presented by Blank et al. [51]. Considering CDM1 and anaerobic conditions, the model was also evaluated by comparison of the predicted essential amino acids with experimental data provided by Armstrong et al. [57]. For this purpose, the lower bound for the particular amino acid’s exchange reaction was set to zero individually, and the essentiality of each amino acid was investigated using FBA by setting the biomass reaction as the objective function. To check the organism’s capability to consume different carbon sources consisting of sucrose, fructose, lactose, and mannose on a complex medium containing yeast extract in aerobic conditions, the organism’s growth was simulated on different carbon sources using FBA. The predicted results were compared with experimental data reported by Pan et al. [58]. The effect of the presence of oxygen on the HA production rate was also studies qualitatively by robustness analysis for HA’s exchange reaction with oxygen’s exchange reaction in comparison with experimental data from Liu et al. [29]. Since the information on the effect of oxygen exchange rate on HA production for a culture medium containing yeast extract and sucrose was available, first, the conditions of this culture medium were simulated. Under these conditions, the oxygen exchange reaction was set to a non-zero value. Then, the HA exchange reaction was set as the objective function and the robustness analysis was performed for the mentioned reaction versus the oxygen exchange reaction.

### 2.3. Comparison of *S. zooepidemicus* and *C. glutamicum* metabolic models for HA biosynthesis

The flux distributions for *S. zooepidemicus* and recombinant *C. glutamicum* metabolic models were calculated to compare central carbon metabolism and HA biosynthetic pathways in these GEMs. For this purpose, first, recombinant *C. glutamicum* was simulated by adding the reaction that simulates HA condensation, and the HA exchange reactions to i*CW773* (as a well-established version of C*. glutamicum* ATCC13032 GEM) [59]. To model an HA-producing C. glutamicum, strategies and pathway modifications detailed in engineering studies, such as Cheng et al. (2019) [20] which utilized *iCW773*, were considered to define the heterologous system. Then, their flux distributions were predicted at a growth rate of 95% of the optimal and for the HA exchange reaction as an objective function using FBA in aerobic conditions. In these simulations, the CDM1 medium was used for the *S. zooepidemicus* model and the CGXII minimal medium was used for recombinant *C. glutamicum* model by setting glucose uptake rates to 18.56 mmol.gDCW^-1^.h^-1^ and 4.67 mmol.gDCW^-1^.h^-1^ respectively. Then, flux distributions were estimated for 100 mmol.gDCW^-1^.h^-1^ glucose consumption rate in each model according to the predicted data by the models. Finally, the flux distributions diagrams for central carbon metabolism and HA biosynthetic pathways for each model were presented for comparison purposes.

Also, *in silico* single- and double-gene deletions for *S. zooepidemicus* in CDM1 medium, and recombinant *C. glutamicum* in CGXII medium were carried out using FBA in aerobic conditions to compare gene interactions in the two networks. Such gene deletion analyses are standard applications of GEMs to understand network robustness and identify essential genes or synthetic lethals [5,60]. Therefore, the percentages of lethal, sick, synthetic lethal, and synthetic sick genes were determined. In single-gene deletion, the relative growth rate (Rgr) was calculated for each gene, which is the ratio of the mutant strain growth rate to the wild strain growth rate. Then, percentages of lethal (Rgr = 0) and sick genes (Rgr < 1) for the two networks were determined. In double-gene deletions, if the predicted growth rate of *in silico* double-mutant strains was less than the growth rate of either of the single-mutant strains, the two genes were considered to have interactions. When the Rgr of double-gene deletions was equal to zero, the two genes were designated as synthetic lethal. Two genes are synthetic sick if their Rgr in double-gene deletion is less than one.

### 2.4. Prediction of strategies for overproduction of HA for the two metabolic models

To predict gene-deletion strategies for overproduction of HA using the S. zooepidemicus GEM, an iterative computational approach based on the Minimization of Metabolic Adjustment (MOMA) principle was employed. The application of this principle, particularly for understanding metabolic rerouting and identifying impactful genetic interventions through iterative single-gene deletion analysis, is supported and contextualized by recent advancements in constraint-based modeling [60]. In this method, the biomass reaction is chosen as the objective function and this MOMA-based analysis is used for iterative single-gene deletion analysis to identify significant genes for increasing HA production rates. The CDM medium in aerobic conditions was considered in these calculations and the glucose consumption rate was set to 18.56 mmol.gDCW^-1^.h^-1^. Single, double and triple gene knockout mutant strains were investigated using the iterative approach: A gene was recognized in round one whose elimination improved the HA production rate and double-mutants were identified by repetition of single-gene deletion analysis in the genetic background of the suitable single-gene mutant. This process was continued until identifying triple-mutant strains with improved HA production rate. If the deletion of a gene in round one represented more than a 40% decrease in the growth rate, it was not allowed to proceed to the next round.

For the recombinant *C. glutamicum* model, MOMA was used for iterative single-gene deletions to determine simulated mutant phenotypes that produced optimal HA production rates. As the method suggested only one gene-deletion, it was used based on multiple optimal solutions [61] to predict candidate reactions that could improve the HA production rate by up- and down-regulation. This approach of leveraging multiple optimal solutions is important as metabolic networks can exhibit flux variability. To this end, 5000 multiple optimal flux distributions using LAMOS [62] of maximized and minimized HA production rate at a constant growth rate of 95% optimal were calculated. Finally, a comparison between multiple optimal solutions of maximized and minimized HA production rates identified the critical reactions to improve the HA production rate in *C. glutamicum*. Advanced algorithms continue to be developed for exploring solution spaces and identifying robust engineering targets [63].

## 3. Results and discussion

### 3.1. The genome-scale metabolic network of *S. zooepidemicus*

*S. zooepidemicus* is a fastidious and facultative anaerobic bacterium. This organism uses the Embden- Meyerhof pathway (EMP) to metabolize glucose for maintenance and cell growth [18]. The complete tricarboxylic acid (TCA) cycle is absent in *S. zooepidemicus*, and this bacterium, in order to grow, needs most amino acids and nucleotides to be present in the growth medium [64]. This auxotrophy is a common characteristic of highly host-adapted bacteria which often lose biosynthetic pathways for compounds readily available from their environment. HA is polymerised from UDP-N-acetylglucosamine (UDP-GlcNAC) and UDP-glucuronic acid (UDP-GlcA) dimers by hyaluronic acid synthase (HAS). The HA glycosyl skeleton originates from glucose. Fructose-6-phosphate is produced from glucose by EMP, then it is converted to glucosamine-6-phosphate, and finally to UDP-GlcNAC. UDP-GlcA, as the other HAS substrate, can also be produced from glucose via the glucuronic acid pathway. The balanced and efficient supply of these activated sugar precursors, UDP-GlcNAc and UDP-GlcA, is a critical control point for both HA yield and molecular weight, as demonstrated in recent metabolic engineering efforts in *S. zooepidemicus* [18,34]. A genome-scale metabolic network of *Streptococcus equi* subsp. *zooepidemicus* ATCC 35246 was reconstructed based on genome annotation and literature data. As the first functional GEM for *S. zooepidemicus*, it was named i*ZN522*. The present model consisted of 677 reactions, 697 metabolites, and 522 genes out of 2049 whole genes (more than 25% coverage). This gene coverage is typical for curated GEMs, focusing on metabolic functions [40]. Out of the 677 chemical reactions in the model, 425 reactions had known genes, 20 reactions were added to the model based on physiological evidence and experimental reports, and 34 reactions were added to the model during the gap-filling process. The model’s features and a comparison of i*ZN522* with other *Streptococci*’s available metabolic models are presented in **Tables 1** and **2**, respectively. Notably, when comparing i*ZN522* with models like that for *S. pyogenes* [65], differences in pathways related to virulence and specific nutrient requirements can become apparent, reflecting their distinct ecological niches.

**Table 1:** An overview of features for S. zooepidemicus metabolic model.

	**Genome (NCBI genome)**	**2049**
**Genes**	i*ZN522* model	522 (25.47%)
	Unknown gene	97
**Reactions**	Exchange	85
	Transport	89
	Balanced	583
	Unbalanced	7
**Metabolites**	Intracellular	593
	Extracellular	104

**Table 2:** Features of i*ZN522* in comparison with other *Streptococci* metabolic models.

Features	Pastink et al. [66]	Levering et al. [67]	i*ZN522* (This study)
Species	*S. thermophilus*	*S. pyogenes*	*S. zooepidemicus*
Reactions	522	576	677
Metabolites	–	558	697
Genes	429	480	522
Total genes	1865	1788	2049
Percent of coverage	23%	26%	25.47%
Percent of reactions without known genes	15%	12%	14%

### 3.2. Evaluation of *iZN522* predictions

Quantitative and qualitative validation approaches were applied to evaluate the accuracy of i*ZN522* predictions. Such validation against experimental data is a cornerstone of establishing confidence in GEM-based simulations [5,38]. One of the quantitative validation steps was predicting the growth rate using a metabolic model in a specific condition. The predicted growth rate by the model in anaerobic conditions and CDM culture medium is close to the reported growth rate by Blank et al. [51]. **Table 3** shows the ability of i*ZN522* to predict the organism’s growth rate in these conditions correctly.

**Table 3:** Comparison of predicted and experimental growth rate.

Culture media	Specific growth rate (h^-1^)		
	Experimental [51]	Predicted (i*ZN522*)	
Anaerobic-CDM	0.4	0.3732	
Error	6.7		Error%=((experimental-predicted)/experimental)*100
Accuracy	93.3		

Furthermore, the prediction of essential amino acids for biomass generation was performed using FBA in CDM1 culture, qualitatively. The results obtained from the metabolic model were compared with experimental amino acid auxotrophy provided by Armstrong et al. [57]. **Table 4** shows that the model predicts the essential amino acids for the organism’s growth with acceptable accuracy, except for the two amino acids lysine and glutamine.

**Table 4:** Predicted essential amino acids using i*ZN522* and comparison with experimental data [57].

Culture media	Experimental	Predicted	Phenotype
CDM1	G	G	TP
CDM1- Asp	G	G	TP
CDM1- Asn	G	G	TP
CDM1- Lys	NG	G	FP
CDM1- Met	NG	NG	TN
CDM1- Thr	G	G	TP
CDM1- Ile	NG	NG	TN
CDM1- Ala	G	G	TP
CDM1- Val	NG	NG	TN
CDM1- Leu	NG	NG	TN
CDM1- Tyr	NG	NG	TN
CDM1- Phe	NG	NG	TN
CDM1- Trp	G	G	TP
CDM1- Ser	G	G	TP
CDM1- Gly	G	G	TP
CDM1- Cys	NG	NG	TN
CDM1- Glu	G	G	TP
CDM1- Pro	G	G	TP
CDM1- Orn	G	G	TP
CDM1- Arg	NG	NG	TN
CDM1- His	NG	NG	TN
CDM1- Gln	NG	G	FP
**Precision**	84.61%		
**Accuracy**	90.90%		
**Sensitivity**	100%		
**Specificity**	81.81%		
**NPV**	100%		
**F-score**	91.66%		

G: growth, NG: not growth, TP: true positive, FP: false positive, TN: true negative, FN: false negative.

Precision = TP/(TP + FP), Accuracy=(TP + TN)/(TP + FP + TN + FN), Sensitivity=(TP/(TP + FN))*100.

Specificity=(TN/(TN + FP))*100, Negative predicted value(NPV)=(TN/(TN + FN))*100,

F-score=2(precision*sensitivity)/(precision+sensitivity).

Indeed, i*ZN522* forecasts the organism’s growth in the absence of lysine and glutamine in contrast to the experimental reports. As metabolic models cannot predict gene-expression data, it may be possible to correct this discordance with experimental data by adding gene-expression data to the model. The integration of omics data, including transcriptomics, is an ongoing effort in the GEM community to enhance model predictivity and address such discrepancies [4,5]. Because the available articles about amino acid consumption in defined culture media are limited, no other evidence was found to explain these results.

For more evaluation, the organism’s growth was qualitatively simulated on various carbon sources such as sucrose, fructose, lactose, and maltose in addition to glucose using FBA to investigate the capability of the model in prediction of the organism’s growth on different carbon sources. The modeling results are presented in **Table 5** and were compared with experimental data reported by Pan et al. [58].

**Table 5:** The prediction of organism’s ability to consume various carbon source using i*ZN522* and comparison with experimental data [58].

Carbon source	Experimental	Predicted	Phenotype
No sugar	NG	NG	TN
Glucose	G	G	TP
Sucrose	G	G	TP
Fructose	G	G	TP
Maltose	G	G	TP
Lactose	G	G	TP
**Precision**	100%		
**Accuracy**	100%		
**Sensitivity**	100%		
**Specificity**	100%		
**NPV**	100%		
**F-score**	100%		

G: growth, NG: not growth, TP: true positive, FP: false positive, TN: true negative, FN: false negative.

Precision = TP/(TP + FP), Accuracy=(TP + TN)/(TP + FP + TN + FN), Sensitivity=(TP/(TP + FN))*100.

Specificity=(TN/(TN + FP))*100, Negative predicted value(NPV)=(TN/(TN + FN))*100,

F-score=2(precision*sensitivity)/(precision+sensitivity).

**Fig 2:**
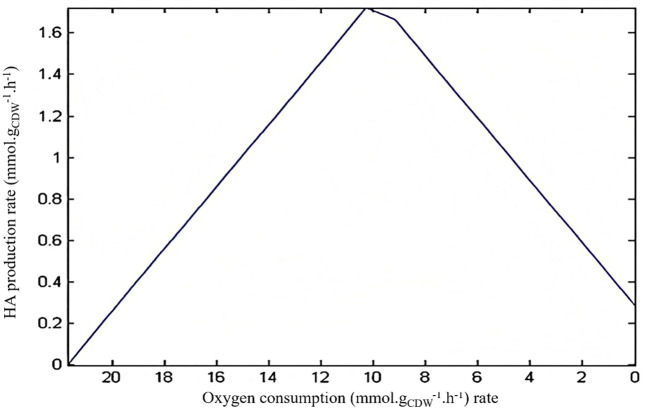
Robustness analysis of hyaluronic acid production under varying oxygen conditions. Graph showing the predicted effect of varying oxygen exchange flux on hyaluronic acid production rate in *S. zooepidemicus* culture media containing yeast extract and sucrose. The robustness curve demonstrates increasing HA flux with increasing oxygen availability, consistent with experimental observations. The x-axis represents oxygen exchange rate (mmol/gDCW/h), and the y-axis represents predicted HA synthesis flux (mmol/gDCW/h). The figure illustrates oxygen’s role as a critical environmental factor enhancing HA biosynthesis in aerobic conditions.

The effect of oxygen presence on the HA production rate was also studied using robustness analysis, qualitatively. Fig 2 shows that oxygen presence leads to an increase in HA production rate following the experimental data presented by Armstrong et al. and Liu et al. [57,68].

### 3.3. Strategies for enhancement of HA production rate in *S. zooepidemicus* using *iZN522*

**Fig 3:**
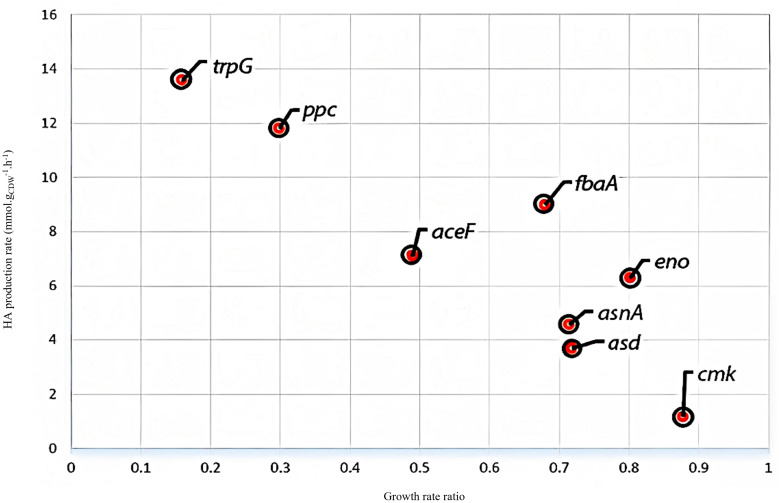
Results of *in silico* single-gene deletion analysis for i*ZN522* metabolic model.

Fig 2, in accordance with experimental data, shows that aerobic conditions are more suitable for the improvement of HA production rate. Thus, aerobic conditions were selected for the prediction of suitable knockout strategies. Then, the maximum growth rate was selected as an objective function, and single-gene deletion analysis was performed using MOMA. The application of MOMA helps identify gene deletions that cause minimal disruption to the overall metabolic flux distribution while achieving a desired phenotypic change [60]. The results of a single genome-wide knockout simulation are illustrated in Fig 3.

The stoichiometric model suggested that the deletion of eight genes, including *trpG*, *ppc*, *aceF*, *fbaA*, *eno*, *asnA*, *asd*, and *cmk*, led to a higher yield of HA production by improving the HA biosynthesis pathway directly and, by decreasing the growth rate, indirectly. Among the eight predicted gene targets, six were removed from further examination: the knockout of *trpG* and *ppc* decreased the growth rate intensively, and deletion of *cmk* did not significantly improve the HA production rate. Furthermore, *aceF*, *fbaA*, and *eno* were vital for the organism’s survival [69].

*AceF* functions as the E2 component of the pyruvate dehydrogenase complex connecting glycolysis to the TCA cycle, playing a critical role in central carbon metabolism. Mutation or deletion of *aceF* has been shown to significantly impact bacterial cytotoxicity and energy metabolism with direct effects on critical secretion systems. The essentiality of *aceF* for cell viability and metabolic regulation has been demonstrated in multiple bacterial species reinforcing its importance in fundamental metabolic flux control. This supports our *in silico* observation of *aceF* essentiality in *S. zooepidemicus* viability and validates its exclusion from knockout candidates [70].

Although our GEM simulations did not predict a significant growth reduction following the in silico deletion of *eno*, *fbaA*, *and* aceF, experimental evidence from other bacteria highlights their critical roles in metabolism, stress tolerance, immune evasion, and virulence. Accordingly, these genes were treated as high-risk knockout targets and excluded from further analysis. Nonetheless, direct validation of their essentiality in *S. zooepidemicus* remains necessary. We propose follow-up experiments including Δ or conditional knockdown strains, in vitro growth assays, oxidative stress and neutrophil killing tests, and complement binding/opsonization/invasion assays for *fbaA* [71,72].

**Fig 4:**
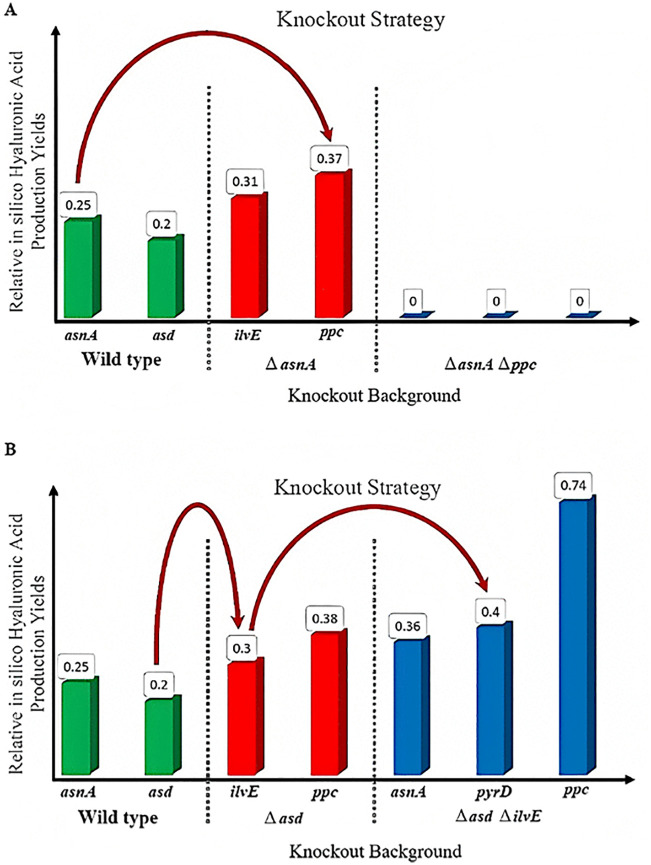
Identifying *in silico* multiple-mutant strain to improve HA production rate using iterative single-gene deletion analysis method: A: *In silico* gene deletions related to the *asnA* (encoding the asparagine synthetase) with the relative predicted HA production yield in moles of HA per mole of glucose B: *In silico* gene deletions related to *asd* (encoding the aspartate semi-aldehyde dehydrogenase) with relative predicted HA production yield in moles of HA per mole of glucose.

Therefore, we consider only *asd* and *asnA* to predict *in silico* double- and triple-mutant strains to improve HA production. First, each of the *asnA* and *asd* genes was removed from the stoichiometric matrix separately. Then, a single-gene deletion analysis was performed again to identify a suitable double- and triple-mutant strain. *In silico* multiple gene-knockout strategies and relative *in silico* HA production yield of mutant strains are presented in Fig 4.

According to Fig 4, the *ilvE*, *ppc*, *pyrD*, and *asnA* genes encode valine transaminase, phosphoenolpyruvate carboxylase, dihydroorotate dehydrogenase, and asparagine synthase, respectively. The omission of these genes as double- and triple-gene deletions can be useful to optimize HA yield.

**Table 6** summarizes the *in silico* iterative single-gene deletion results and their HA production rate. The mutant strain growth rate ratio to wild strain growth rate for each simulated mutant strain is presented in **Table 6**.

**Table 6:** Predicted growth rate and HA production rate in single, double, and triple *S. zooepidemicus in silico* mutant strains.

Knockout construct	Predicted growth rate of parental	Predicted HA production rate (mmol.gCDW^-1^.h^-1^)
None	1	0
***Single knockout***		
*asnA*	0.71	4.58
*asd*	0.72	3.71
***Double knockouts***		
*asnA,ilvE*	0.33	5.77
*asnA,ppc*	0.30	6.98
*asd,ilvE*	0.37	5.5
*asd,ppc*	0.31	7.04
***Triple knockouts***		
*asd,ilvE,asnA*	0.37	6.66
*asd,ilvE,pyrD*	0.51	7.33
*asd,ilvE,ppc*	0.31	13.67

All mutant strains increase the HA production rate by decreasing the growth rate and increasing hyaluronan synthase availability to their precursors. This inverse relationship between growth and production of secondary metabolites or complex polymers like HA is a common challenge in metabolic engineering, often requiring strategies that decouple growth from production [8].

**Fig 5:**
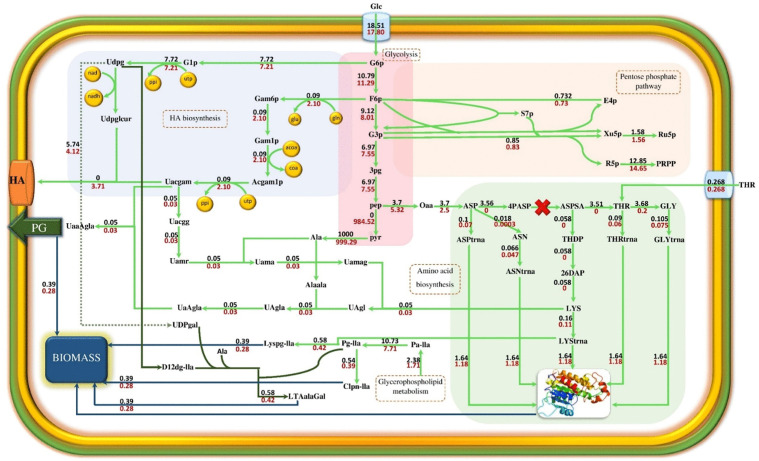
The metabolic flux distribution in ∆*asd* strain in comparison with the wild strain. **Black values indicate the reaction fluxes of the wild strain, and red values indicate reaction fluxes of ∆*asd* strain.** The unit of fluxes is mmol.gDCW^-1^.h^-1^ The multiplication sign indicates the deletion of a gene. The equivalent abbreviations for important metabolites are given below (Udpg: UDP-glucose, Uacgam: UDP-N-acetyl glucosamine, PG: Peptidoglycan, Clpn-lla: Cardiolipin, Lyspg-lla: Lysyl phosphatidylglycerol, LTAalaGal: Lipoteichoic acid derivatives, HA: Hyaluronic acid).

Finally, the triple-mutant strain Δ*asd* Δ*ilvE* Δ*pyr* was selected as the most efficient mutant strain due to its suitable growth rate and high HA production rate. The comparison of flux distribution for the wild strain with the mentioned triple-mutant strain has demonstrated the effectiveness of eliminating three genes, including *asd*, *ilvE*, and *pyrD* on HA production metabolic pathway. Fig 5 illustrates the flux distributions of metabolic pathways after *asd* gene deletion. The *asd* encodes aspartate semi-aldehyde dehydrogenase and is involved in the transformation of aspartate to threonine and lysine.

**Fig 6:**
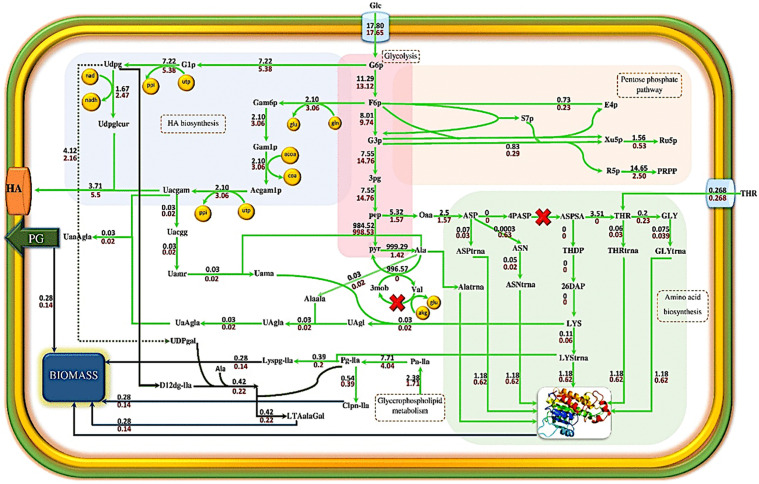
Comparison of metabolic flux distribution in ∆*asd* ∆*ilvE* strain with ∆*asd* strain. **Red values refer to the reaction fluxes of the *ilvE* ∆*asd* strain.** The fluxes are reported in mmol.gCDW^-1^.h^-1^. (Udpg: UDP-glucose, Uacgam: UDP-N-acetylglucosamine, PG: peptidoglycan, Clpn-lla: cardiolipin, Lyspg-lla: lysyl phosphatidylglycerol, LTAalaGal: lipoteichoic acid derivatives, HA Hyaluronic acid, 3 mob: 3-methyl-2-oxobutanate, akg: alpha-ketoglutarate, glu: glutamate).

The elimination of the *asd* decreases the flux of reactions that produce aspartate-tRNA, threonine-tRNA, glycine-tRNA, lysine-tRNA, and asparagine-tRNA and led to reducing the flux of protein formation as part of the biomass from 1.64 in wild-type to 1.18 in ∆*asd*. Lysine is involved in the formation of cardiolipin, lipoic acid, lysyl phosphatidylglycerol, and peptidoglycan (as parts of biomass composition). When the aspartate to lysine conversion pathway is blocked, the flux passing through the enzymatic reactions mentioned is limited, and the growth rate decreases from 0.39 to 0.28 per hour. *ilvE* was identified as the second knockout for enhancement of HA production rate, which encodes the valine transaminase enzyme. Valine transaminase produces 3-methyl-2-oxobutanate by consuming the valine and converting one alpha-ketoglutarate to glutamine. Alanine is converted to pyruvate simultaneously. Finally, the pyruvate regenerates alanine by alanine transaminase. According to Fig 6, a cycle is formed in the ∆*asd* strain, which continuously produces alanine. Elimination of *ilvE* from the stoichiometric matrix blocks the cycle transforming pyruvate to alanine. Thus, the alanine-transaminase flux decreases from 999.29 mmol.gDCW^-1^.h^-1^ in ∆*asd* strain to 1.42 mmol.gDCW^-1^.h^-1^ in ∆*asd* ∆*ilvE* strain. Whereas alanine is involved in the biosynthesis of cardiolipin, peptidoglycan, and lipoteichoic acid, which generate a major part of biomass, the growth rate decreases from 0.28 h^-1^ in ∆*asd* to 0.14 h^-1^ in ∆*asd* ∆*ilvE* by reduced alanine production.

Hyaluronic acid is synthesized in the two main branches of the intermediate compounds of the glycolysis pathway. The first branch originates from glucose-6-phosphate, which forms UDP-glucuronic acid by consuming one UTP and NAD molecules. Fructose-6-phosphate is the origin of the second branch, which is converted to UDP-N-acetylglucosamine by consuming one molecule of UTP, one molecule of glutamine, and acetyl CoA. Finally, the precursors UDP-N-acetylglucosamine and UDP- glucuronic acid bind together to form the HA chain by the hyaluronan synthase enzyme. UDP-glucose and UDP-N-acetylglucosamine are precursors of HA production. Furthermore, they are involved in the synthesis of cardiolipin, peptidoglycan, and lipoteichoic acid (Fig 5). Thus, when *asd* and *ilvE* genes are eliminated from the metabolic model, growth rate decreases, and more HA precursors are consumed for the HA biosynthesis pathway. As a result, hyaluronan synthase flux increases from zero in the wild strain to 5.5 mmol.gDCW^-1^.h^-1^ in ∆*asd* ∆*ilvE.pyrD*, which as the third identified knockout for improvement of HA biosynthesis, encodes dihydroorotate dehydrogenase, participates in oxidation of (*S*)-dihydroorotate to orotate and in the *de novo* biosynthesis of pyrimidine nucleotides. The deletion of *pyrD* increases both growth rate and HA production rates, simultaneously. On the other hand, in the ∆*asd* ∆*ilvE* ∆*pyrD* triple-mutant strain, HA production and growth rate are coupled together. The omission of *pyrD* blocks the biosynthesis of UMP from aspartate. Thus, UMP is provided by nucleotide salvage pathways in the triple-mutant strain. In the triple-mutant strain, the flux of reaction related to phosphoribosyl pyrophosphate synthetase (PRPP synthetase) increases compared with ∆*asd* ∆*ilvE*.

The improved flux of PRPP synthetase activates the associated reaction with the uracil phosphoribosyltransferase (UPRT) enzyme in the ∆*asd* ∆*ilvE* ∆*pyrD* strain. UPRT consumes one unit of uracil and 5-phosphoribosyl-1-pyrophosphate to produce a unit of UMP. Also, when the reaction of dihydroorotate dehydrogenase is inactivated, a cycle is created that continuously provides the UTP required to produce UDP-N-acetylglucosamine and UDP-glucose (similar precursors for HA and biomass production). Therefore, biomass and HA production are not limited. Furthermore, when the flux of reactions associated with the production of ribonucleotides and deoxyribonucleotides increases, the RNA and DNA production fluxes which form part of the biomass, enhance compared to the double-mutant strain. Thus, the growth rate and HA production rate increase from 0.15 h^-1^ and 5.5 mmol.gDCW^-1^.h^-1^ in ∆*asd* ∆*ilvE* strain to 0.20 h^-1^ and 7.33 mmol.gDCW^-1^.h^-1^ in the ∆*asd* ∆*il*vE ∆*pyrD*, respectively. These predicted improvements highlight the potential of systematic multi-gene knockout strategies identified via GEMs, although experimental validation of such complex mutant phenotypes is essential [5].

### 3.4. Strategies for enhancement of HA production rate in recombinant *C. glutamicum* using i*CW773*

**Fig 7:**
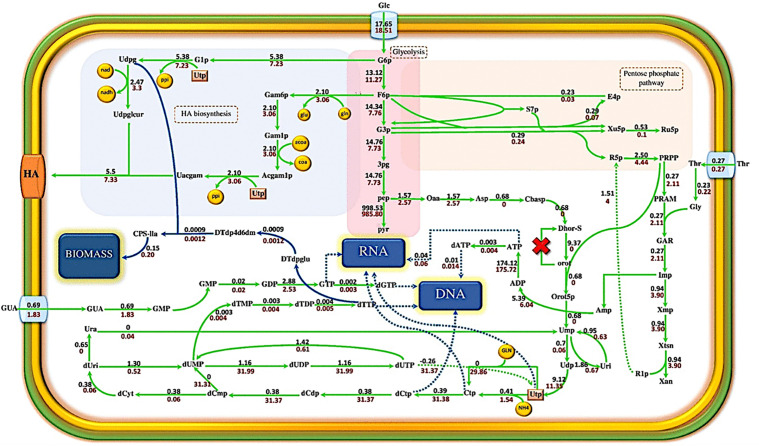
Comparing the metabolic flux distribution in the ∆*asd* ∆*ilvE* ∆*pyrD* strain with the ∆*asd* ∆*ilvE* strain. The black values represent the reaction fluxes of the ∆*asd* ∆*ilvE* strain. Red values refer to the reaction fluxes of the ∆*asd* ∆*ilvE* ∆*pyrD* strain. The fluxes are reported in mmol.gCDW^-1^.h^-1^. The cross indicates the deletion of the gene. (Udpg: UDP-glucose, Uacgam: UDP-N-acetyl glucosamine, PG: peptidoglycan, CPS-lla: polysaccharide units, Ura: uracil, Uri: uridine, GUA: guanine).

At first, an HA disaccharide binding reaction and an HA exchange reaction were added to i*CW773*. Then single-gene deletion analysis by MOMA was performed in aerobic conditions for CGXII minimal culture medium with a glucose consumption rate of 4.67 mmol.gDCW^-1^.h^-1^. Three genes, including *eno*, *nadhd*, and *zwf,* were suggested as genes whose knockouts improved the HA production rate, as shown in Fig 7.

**Fig 8:**
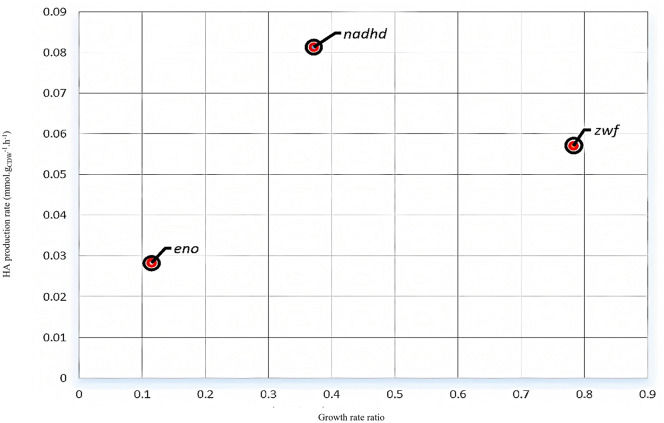
Results of *in silico* single-gene deletion analysis for i*CW773* metabolic model.

The *nadhd*, *eno*, and *zwf* genes encode the NADH dehydrogenase, enolase, and glucose-6-phosphate dehydrogenase, respectively. As the lack of *eno* and *nadhd* genes, decrease the growth rate by less than 40 percent, the only suitable gene deletion is that of the *zwf* gene, which increases the HA production rate to 0.05 mmol.gDCW^-1^.h^-1^. The *zwf* gene encodes the enzyme glucose-6-phosphate dehydrogenase, which initiates the oxidative pentose phosphate pathway [73]. The elimination of this gene leads to more glucose-6-phosphate entering the HA biosynthetic pathway. This strategy of blocking the initial step of the PPP to channel carbon towards a desired product is a common approach in metabolic engineering of various microorganisms [21]. In addition, the growth rate is reduced by 0.78 percent of the wild strain’s growth rate by attenuation of the generation of NADPH in the oxidative pentose phosphate pathway, which is used for anabolic reactions. Thus, *zwf* knock out amplifies the flux of hyaluronan synthase by redirecting carbon flux from biomass to HA generation (Fig 8). This result is in accordance to experimental data presented by Cheng et al. [20].

In our *in silico* gene deletion experiments, we classified genes based on their impact on growth. ‘Lethal’ genes denote those whose deletion completely halts growth, indicating essentiality under modeled conditions. ‘Sick’ genes cause a significant but not fatal growth decrease, reflecting partial impairment. ‘Synthetic lethal’ pairs describe gene combinations where simultaneous deletion is lethal even though single deletions are not, highlighting compensatory pathways. ‘Synthetic sick’ pairs involve combined deletions leading to more severe growth defects than isolated knockouts. These categorizations enable precise characterization of network robustness and pinpoint promising targets for metabolic engineering.

We identified efficient double- and triple-gene knockouts in *S. zooepidemicus* to optimize HA production rate by iterative MOMA single-gene deletion analysis approach. However, our attempts at the above approach to identify efficient *in silico* multiple-mutant strain in the *C. glutamicum* model (i*CW773*) to produce an optimal HA production rate were not successful. It seems that the difference in gene interactions in the two metabolic networks has caused this difference. To prove this assumption, single-gene deletion analysis, double-gene deletion analysis with one, and at least five gene interactions were performed. Thus, the percentage of lethal and sick genes for the two metabolic networks i*CW773* and i*ZN522* were obtained. In addition, synthetic lethal and synthetic sick genes with one and at least five interactions for the two models were calculated and compared. According to **Table 7**, the percentage of lethal genes, synthetic lethal genes with one interaction, and at least five interactions for the i*ZN522* model are much higher than the amount calculated for the i*CW773* model. This suggests a potentially higher degree of metabolic network robustness or redundancy in the i*CW773* model of *C. glutamicum* compared to i*ZN522* for *S. zooepidemicus*, which could explain the differential success of iterative single-gene deletion strategies for complex product optimization [60].

**Table 7:** Results of single- and double-gene deletion analysis *Streptococcus zooepidemicus* and recombinant *Corynebacterium glutamicum* with flux balance analysis approach.

Percent of genes						
Bacteria	Lethal	Sick (GR < 1)	Synthetic lethal with interaction	Synthetic sick (GR < 1) with interaction	Synthetic lethal with ≥5 interactions	Synthetic sick (GR < 1) with≥5 interaction
*Streptococcus* *zooepidemicus*	31.61%	11.30%	0.027%	0.006%	0.05%	0.72%
*Corynebacterium* *glutamicum*	8.8%	13.58%	0.014%	0.01%	0.017%	0.21%

**Fig 9:**
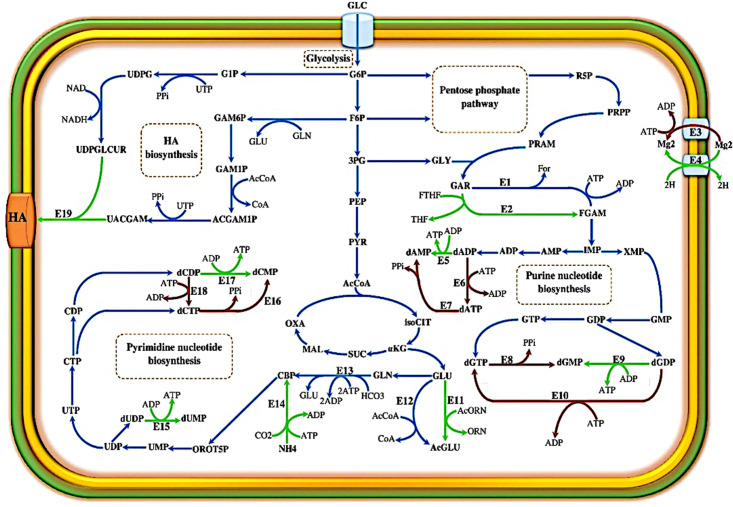
Providing solutions to optimize the HA production rate based on multiple optimal solutions in the metabolic model of i*CW773.* The blue arrows represent reactions that carry a normal flux; the green arrows should up-regulate, while the flux of reactions with the red arrows should down-regulate.

The results indicate that there are more target points in i*ZN522* than i*CW773* to reduce the growth rate. Thus, the iterative single-gene deletions strategy will not be sufficient for HA optimization in this strain. Furthermore, a method based on multiple optimal solutions was used to improve HA production using the i*CW773* metabolic network. To identify critical reactions that play an influential role in the enhancement of HA production under recombinant *C. glutamicum*, 5000 multiple optimal flux distributions using LAMOS in two conditions at a constant growth rate of 95% optimal were calculated. Then, the comparison of multiple optimal solutions of maximized and minimized HA production rate under recombinant *C. glutamicum* was carried out to identify target reactions that should be up- or down-regulated to optimize the HA production rate. According to model predictions, the up-regulation of reactions of phosphoribosylglycinamide formyltransferase, Mg antiporter, adenylate kinase, deoxy guanylate kinase, glutamate N-acetyltransferase, carbamate kinase, uridylate kinase, cytidylate kinase, hyaluronan synthase and down-regulation of reactions of GAR transformylase, Mg^2+^ ABC transporter, nucleoside diphosphate kinase, and nucleoside triphosphate pyrophosphorylase increase HA production rate from zero to 0.22 mmol.gDCW^-1^.h^-1^ (Fig 9).

The model predicts that upregulation of anabolic reactions responsible for precursor biosynthesis coupled with downregulation of ATP-consuming processes may collectively promote increased hyaluronic acid production, suggesting focused metabolic flux control as a promising strategy [8].

For the production of each unit of disaccharide in the HA chain, five molecules of ATP are consumed [33]. Therefore, the above changes reduce the energy cost of producing the metabolites which are essential to generate biomass, and the excess ATP is used to increase the HA production rate. In addition, for each entry of an Mg^2+^ ion, two proton ions are transferred to the extracellular environment by the Mg^2+^ antiporter. Assuming the transient flux of the Mg^2+^ antiporter enhances, the proton gradient in the extracellular environment increases, consequently increasing the flux of ATP synthase and ATP production. The method based on multiple optimal solutions suggested that the up-regulation of the glutamate-N-acetyltransferase reaction can also enhance the HA production rate. Acetylglucosamine is a precursor required for the formation of HA and biomass. Therefore, saving acetyl-CoA can also increase HA. In fact, increasing the reaction flux of glutamate-N-acetyltransferase, which converts a unit of acetyl ornithine to ornithine, provides the acetyl that is needed to produce acetyl glutamate. Thus, the acetyl-CoA required to increase HA production is maintained and improves the HA production rate.

### 3.5. The comparison of HA production pathway in i*ZN522* and i*CW773*

**Fig 10:**
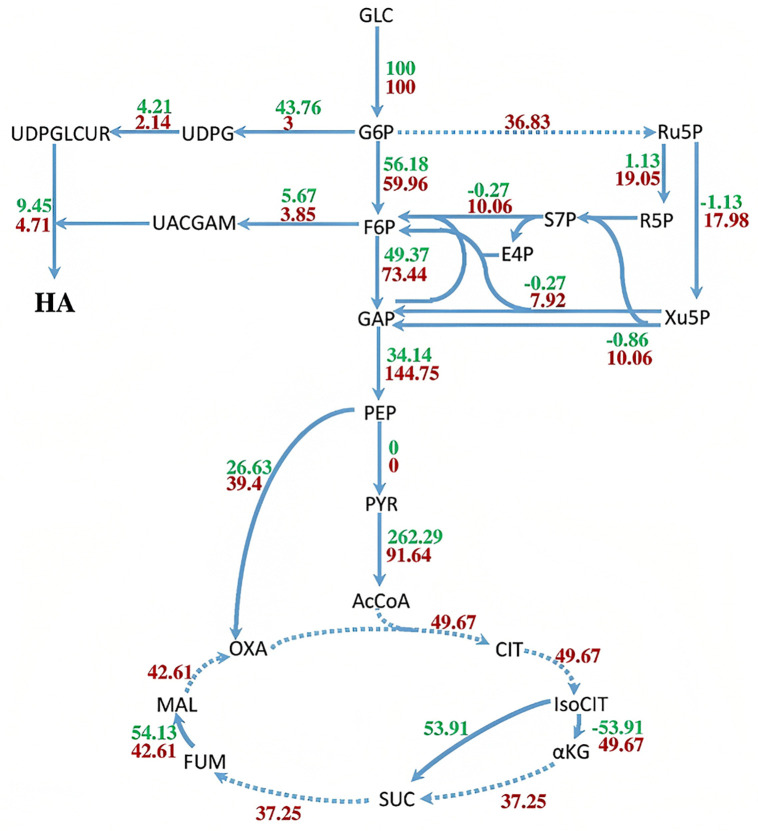
The Comparison of flux distribution of central carbon metabolism pathway and HA production pathway in i*ZN522* and i*CW773* metabolic networks. *S. zooepidemicus* fluxes were reported in green, and recombinant *C. glutamicum* fluxes were shown in red. The dot line also indicates reactions that are present only in *C. glutamicum*, and *S. zooepidemicus* lacks them. Negative fluxes also indicate the reaction flux in the opposite direction.

For this purpose, the flux distribution for HA production, especially in central carbon metabolism (glycolysis, pentose-phosphate, and Krebs cycle), was calculated for both strains using FBA with a glucose consumption rate of 100 mmol.gDCW^-1^.h^-1^. These flux distributions were compared to identify further strategies for improving HA production in recombinant *C. glutamicum*. In contrast to *S. zooepidemicus*, the oxidative pentose-phosphate pathway is active in *C. glutamicum*, as shown in Fig 10.

During this process, glucose-6-phosphate is first converted to 6-phosphogluconate and produces 1 mole of NADPH by the glucose-6-phosphate dehydrogenase. The enzyme 6-phosphogluconate dehydrogenase then produces ribulose-5-phosphate and releases 1 unit of CO_2_ and NADPH from the 6-phosphogluconate. As *C. glutamicum* has the potential to grow in a minimal culture medium, it needs higher volumes of NADPH to produce the amino acids, cofactors, and nucleotide compounds that are necessary for its growth. This inherent metabolic capability for NADPH generation is a hallmark of *C. glutamicum*’s robust central metabolism, often exploited in metabolic engineering [21,22]. *S. zooepidemicus* is a fastidious bacterium that can only grow in complex culture media, so it requires less NADPH than *C. glutamicum* due to its defective amino acid biosynthesis pathways. The oxidative pentose-phosphate pathway in *C. glutamicum* causes a large volume of glucose-6-phosphate to enter this pathway. Thus, less glucose-6-phosphate enters the HA production pathway. Hence the enzyme hyaluronan synthase in *C. glutamicum* carries a lower flux than in *S. zooepidemicus* under baseline non-engineered conditions for HA production. The non-oxidative pentose-phosphate pathway contains transketolase and transaldolase activity. In *S. zooepidemicus*, these enzymes use fructose-6-phosphate and glyceraldehyde-3-phosphate to provide ribose-5-phosphate needed for nucleotides production. In *S. zooepidemicus*, a part of the fructose-6-phosphate and glyceraldehyde-3-phosphate produced in the glycolysis pathway is used to produce ribose-5-phosphate, so the flux passing through both reactions of phosphofructokinase and glyceraldehyde-3-phosphate dehydrogenase is reduced. Whereas, in *C. glutamicum*, non-oxidative reactions of pentose-phosphate pathway are carried out to convert ribose-5-phosphate and xylulose-5-phosphate to fructose-6-phosphate and glyceraldehyde-3-phosphate, which increases the fluxes of phosphofructokinase and glyceraldehyde-3-phosphate dehydrogenase. This difference in the pentose-phosphate pathway depends on the activation or inactivation of glucose-6-phosphate dehydrogenase. Thus, the omission of the glucose-6-phosphate dehydrogenase gene can improve the HA production rate in recombinant *C. glutamicum*. Most of the Krebs cycle reactions in *S. zooepidemicus* were absent. In contrast, recombinant *C. glutamicum* has a complete Krebs cycle and electron transport chain. Therefore, in recombinant *C. glutamicum*, more ATP is produced per mole of glucose than in *S. zooepidemicus*. According to model results, the amount of ATP produced per mole of glucose in *S. zooepidemicus* is equivalent to 20 molecules of ATP, but in *C. glutamicum* it is equal to 26.58 molecules of ATP. The HA biosynthesis pathway consumes ATP; therefore, considering the ATP yield in both models, recombinant *C. glutamicum* appears theoretically more suitable for HA production from an energetic standpoint, provided carbon flux can be efficiently channeled [8].

This comprehensive comparison using GEMs reveals distinct metabolic architectures and energetic efficiencies that underpin the different natural and engineered capacities for HA production in these two organisms. Such insights are crucial for selecting appropriate host chassis and devising rational engineering strategies for improved biopolymer synthesis [5,37]. Further developments, such as incorporating kinetic information or using hybrid modeling approaches [74], could offer even deeper understanding and more precise predictions for optimizing HA production.

### 3.6. Comparative discussion on key essential genes across organisms

We elaborate on critical enzymatic functions shared by *S. zooepidemicus* and *C. glutamicum*, emphasizing genes such as *asd* (aspartate semi-aldehyde dehydrogenase) and *asnA* (asparagine synthase), which are central to amino acid biosynthesis. These enzymes influence the pool of metabolites that serve dual roles in biomass formation and hyaluronic acid precursor synthesis. The strategic modulation of these genes markedly affects carbon flux toward HA biosynthesis pathways, demonstrating their high value as engineering candidates across both models.

In this study, the i*ZN522* metabolic model was reconstructed as the first metabolic model for *S. zooepidemicus*. These flux perturbations support a balanced allocation of resources between biomass synthesis and hyaluronic acid production, thereby optimizing overall cellular productivity under engineering conditions. This model was utilized to identify the parameters influencing HA production and to explore potential solutions for enhancing HA production through targeted genetic modifications. Furthermore, the metabolic model of recombinant *C. glutamicum* was simulated as a suitable recombinant strain for HA production. Strategies involving up- and down-regulation of key reactions, guided by computational analysis, were also proposed to enhance HA production in this strain were also proposed. Lastly, a comparison of the central carbon metabolic pathways in both strains elucidated the factors contributing to the higher HA production potential and different metabolic efficiencies in *S. zooepidemicus* compared to the baseline engineered recombinant *C. glutamicum*.

The development of *i*ZN522 offers a significant advancement for understanding and engineering *S. zooepidemicus*, a key industrial HA producer. The model-driven identification of gene knockout targets provides specific, actionable hypotheses for experimental validation aimed at increasing HA titers and productivity [8,18]. The insights gained from the comparative analysis with *C. glutamicum* not only explain observed differences in HA production capabilities but also inform broader strategies for host selection and optimization in metabolic engineering [37].

Looking forward, the *i*ZN522 model can serve as a foundational tool for more advanced studies. Future efforts could focus on its expansion by integrating multi-omics data (e.g., transcriptomics, proteomics) to capture regulatory dynamics and further refine predictions [4,5]. Investigating the metabolic determinants of HA molecular weight control, a critical quality attribute, represents another important avenue where *i*ZN522 could provide valuable insights, potentially guiding engineering strategies similar to those explored recently [34]. Moreover, the development of dynamic or hybrid models that incorporate kinetic parameters and fermentation process variables could lead to more accurate simulations of industrial bioprocesses [63,74]. Continued experimental validation of the targets identified in this study will be paramount. Ultimately, this work contributes to the growing body of knowledge in systems metabolic engineering, paving the way for more efficient and sustainable biotechnological production of HA and other valuable biopolymers [12,15].

## 4. Conclusion

In this study, we report the first comprehensive genome-scale metabolic model of *Streptococcus zooepidemicus* (i*ZN522*), providing strong quantitative validation against experimental growth data and substrate utilization profiles. Comparative model-based analyses with the recombinant *Corynebacterium glutamicum* model (i*CW773*) reveal distinct metabolic engineering strategies for increased hyaluronic acid production: targeted gene knockouts in *S. zooepidemicus* versus flux modulation in *C. glutamicum*. These insights offer a systems-level understanding that bridges native and recombinant hosts, guiding rational strain improvement for industrial HA biosynthesis.

## Supporting information

S1 File Supporting information file. (DOCX)
