# Supplementary material for: Reconstruction of a genome-scale metabolic model for Streptococcus zooepidemicus: Comparison with Corynebacterium glutamicum to study hyaluronic acid production
Source: PLoS One. 2025 Dec 31;20(12):e0335509. doi: 10.1371/journal.pone.0335509 (PMC12755811; doi:10.1371/journal.pone.0335509)
Supplement: S1 File — (DOCX) [file pone.0335509.s001.docx]

**Supplementary file S1**

**Reconstruction of a genome-scale metabolic model for Streptococcus zooepidemicus: comparison with *Corynebacterium glutamicum* to study hyaluronic acid production**

Zahra Nikuiyan^1^, Fatemeh Tabandeh^1*^, Ehsan Motamedian^2*^, Saeed Salehi^2,#a^, Marjan Talebi^3,4^, Rouzbeh Almasi Ghale^1^

^1^Department of Energy and Environmental Biotechnology, National Institute of Genetic Engineering and Biotechnology (NIGEB), 14965/161, Tehran, Iran.

^2^Department of Biotechnology, Faculty of Chemical Engineering, Tarbiat Modares University, 4838-14155, Tehran, Iran.

^3^Department of Pharmacognosy, TeMS.C., Islamic Azad University, Tehran, Iran.

^4^Herbal Pharmacology Research Center, TeMS.C., Islamic Azad University, Tehran, Iran

^#a^Current Address: R&D Department, Maya Zist Farayand Co., Tehran, Iran.

*Corresponding authors: taban_f@nigeb.ac.ir (F.T.), ORCID ID: 0000-0002-4989-7598; motamedian@modares.ac.ir (E.M.)

**S1 Table. Constraints applied to simulate defined culture media.**

| **Metabolite** | **Exchange reaction** | **Lower bound (mmol.grCDW^-1^.h^-1^)** | |
| --- | --- | --- | --- |
|  |  | **Anaerobic CDM** | **CDM1** |
| **Para-aminobenzoate** | EX_4abz_e | -1000 | -1000 |
| **acetate** | EX_ac_e | 0 | 0 |
| **Adenine** | EX_ade_e | -10 | -10 |
| **Alanine** | EX_ala_L_e | -0.104 | -0.104 |
| **Arginine** | EX_arg_L_e | -0.078 | -0.078 |
| **Asparagine** | EX_asn_L_e | -0.047 | -0.047 |
| **Aspartic acid** | EX_asp_L_e | -0.016 | -0.016 |
| **Biotin** | EX_btn_e | -10 | -10 |
| **CO2** | EX_co2_e | 0 | 0 |
| **Cytosine** | EX_csn_e | 0 | 0 |
| **Cysteine** | EX_cyn_e | -10 | 0 |
| **Cysteine** | EX_cys_L_e | -1.84 | -1.84 |
| **Ethanol** | EX_etoh_e | 0 | 0 |
| **Folate** | EX_fol_e | -10 | -10 |
| **Formate** | EX_for_e | 0 | 0 |
| **Glucose** | EX_glc_e | -18.51 | -18.51 |
| **Glutamine** | EX_gln_L_e | -0.822 | -0.822 |
| **Glutamic acid** | EX_glu_L_e | -10 | -10 |
| **Glycine** | EX_gly_e | -0.09 | -0.09 |
| **Guanine** | EX_gua_e | -10 | -10 |
| **H^+^** | EX_h_e | -1000 | -1000 |
| **HCO_3_^-^** | EX_hco3_e | 0 | 0 |
| **Histidine** | EX_his_L_e | -0.177 | -0.177 |
| **Isoleucine** | EX_ile_L_e | -0.182 | -0.182 |
| **Lactate** | EX_lac_L_e | 0 | 0 |
| **Leucine** | EX_leu_L_e | -0.386 | -0.386 |
| **Lysine** | EX_lys_L_e | -0.15 | -0.15 |
| **Methionine** | EX_met_L_e | -0.154 | -0.154 |
| **Nicotinate** | EX_nac_e | -10 | -10 |
| **Ammonia** | EX_nh3_e | 0 | 0 |
| **Ammonium** | EX_nh4_e | 0 | -1000 |
| **Nitrite** | EX_no2_e | 0 | 0 |
| **Nitrate** | EX_no3_e | -1000 | 0 |
| **O2** | EX_o2_e | 0 | 0 |
| **Ornithine** | EX_orn_e | 0 | -1000 |
| **Orotate** | EX_orot_e | 0 | 0 |
| **Phenylalanine** | EX_phe_L_e | -0.108 | -0.108 |
| **Phosphate** | EX_pi_e | -1000 | -1000 |
| **Pantothenate** | EX_pnto_R_e | -10 | -10 |
| **Proline** | EX_pro_L_e | -0.06 | -0.06 |
| **Pyrodoxamine** | EX_pydam_e | -10 | -10 |
| **Pyrodoxine** | EX_pydxn_e | -10 | -10 |
| **Riboflavin** | EX_ribflv_e | -10 | -10 |
| **Serine** | EX_ser_L_e | -0.194 | -0.194 |
| **Sulfate** | EX_so4_e | -1000 | -1000 |
| **Succinate** | EX_succ_e | 0 | 0 |
| **Thiamin** | EX_thm_e | -10 | -10 |
| **Threonine** | EX_thr_L_e | -0.268 | -0.268 |
| **Thymidine** | EX_thymd_e | 0 | 0 |
| **Tryptophan** | EX_trp_L_e | -0.022 | -0.022 |
| **Tyrosine** | EX_tyr_L_e | -0.086 | -0.086 |
| **Uracil** | EX_ura_e | -10 | -10 |
| **Valine** | EX_val_L_e | -0.231 | -0.231 |
| **Xanthine** | EX_xan_e | 0 | 0 |
| **Zn^2+^** | EX_zn2_e | -1000 | -1000 |
| **Mn^2+^** | EX_mn2_e | -1000 | -1000 |
| **Ca^2+^** | EX_ca2_e | -1000 | -1000 |
| **Cl^-^** | EX_cl_e | -1000 | -1000 |
| **K^+^** | EX_k_e | -1000 | -1000 |
| **Na^+^** | EX_na1_e | -1000 | -1000 |
| **Mg^2+^** | EX_mg2_e | -1000 | -1000 |
| **Fe^2+^** | EX_fe2_e | -1000 | -1000 |
| **Fe^3+^** | EX_fe3_e | -1000 | -1000 |
| **Cu^2+^** | EX_cu2_e | -1000 | -1000 |
| **Cyanocobalamin** | EX_cyncblm_e | -10 | -10 |
| **Hydroxylproline** | EX_hpro_e | 0 | 0 |
| **H_2_S** | EX_h2s_e | 0 | 0 |

**S2 Table. The CDM and CDM1 culture media composition.**

| **CDM** | | **CDM1** | |
| --- | --- | --- | --- |
| **Component** | **Concentration (mg/L)** | **Component** | **Concentration (mg/L)** |
| FeSO_4_.7H_2_O | 5 | Na_2_HPO.12H_2_O | 1500 |
| Fe(NO_3_)_2_.9H_2_O | 1 | FeNH_4_(SO_4_)_2_.12H_2_O | 22 |
| K_2_HPO_4_ | 200 | ZnSO_4_.7H_2_O | 0.6 |
| KH_2_PO_4_ | 1000 | KH_2_PO_4_ | 500 |
| MgSO_4_.7H_2_O | 700 | MgSO_4_.7H_2_O | 500 |
| MnSO_4_ | 5 | MnCl_2_.4H_2_O | 5 |
| DL-Alanine | 100 | L-Alanine | 100 |
| L-Arginine | 20 | L-Arginine | 100 |
| L-Aspartic acid | 100 | L-Aspartic acid | 100 |
| L-Cystine | 50 | Ornithine | 100 |
| L-Glutamic acid | 100 | L-Glutamic acid | 100 |
| L-Glutamine | 200 | L-Glutamine | 5600 |
| Glycine | 100 | Glycine | 100 |
| L-Histidine | 100 | L-Histidine | 100 |
| L-Isoleucine | 100 | L-Isoleucine | 100 |
| L-Leucine | 100 | L-Leucine | 100 |
| L-Lysine | 100 | L-Lysine | 100 |
| L-Methionine | 100 | L-Methionine | 100 |
| L-Phenylalanine | 100 | L-Phenylalanine | 100 |
| L-Proline | 100 | L-Proline | 100 |
| Hydroxy-L-proline | 100 | - | - |
| L-Serine | 100 | L-Serine | 100 |
| L-Threonine | 200 | L-Threonine | 100 |
| L-Tryptophan | 100 | L-Tryptophan | 100 |
| L-Tyrosine | 100 | L-Tyrosine | 100 |
| L-Valine | 100 | L-Valine | 100 |
| Para-Aminobenzoic acid (PABA) | 0.2 | Para-Aminobenzoic acid | 0.1 |
| Biotin | 0.2 | Biotin | 0.01 |
| Folic acid | 0.8 | Folic acid | 0.1 |
| Niacinamide | 1 | - | - |
| Beta-Nicotinamide adenine dinucleotide | 2.5 | Nicotinamide | 2 |
| Pantothenate calcium salt | 2 | Pantothenate | 0.8 |
| Pyridoxal | 1 | Pyridoxal | 1 |
| Pyridoxamine dihydrochloride | 1 | Pyridoxamine | 0.8 |
| Riboflavin | 2 | Riboflavin | 0.4 |
| Thiamine hydrochloride | 1 | Thiamine hydrochloride | 0.4 |
| Vitamin B12 | 0.1 | Cyanocobalamin | 0.1 |
| Glucose | 5000 | Glucose | 20000 |
| Adenine | 20 | Adenine | 30 |
| Guanine hydrochloride | 20 | Guanine | 30 |
| Uracil | 20 | Uracil | 30 |
| CaCl_2_.6H_2_O | 10 | CaCl_2_.2H_2_O | 50 |
| NaC_2_H_3_O_2_.3H_2_O | 4500 | - | - |
| L-Cysteine | 500 | L-Cysteine | 100 |
| NaHCO_3_ | 2500 | - | - |
| NaH_2_PO_4_.H_2_O | 3195 | - | - |
| Na_2_HPO_4_ | 7350 | Na_2_HPO_4_.12H_2_O | 1500 |
| - | - | CuSO_4_.5H_2_O | 0.6 |

**S3 Table. The complex medium composition.**

| **Component** | **Concentration (g/L)** |
| --- | --- |
| Glucose* | 30 |
| Yeast extract | 30 |
| K_2_HPO_4_ | 2.5 |
| NaCl | 2 |
| MgSO_4_.7H_2_O | 1.5 |

*Glucose in the complex medium was replaced with four other sugars such as sucrose, fructose, lactose, and maltose.

**Formulation of DNA formation reaction**

The molar ratio of DNA per gram of biomass is used as the stoichiometric coefficient of DNA in the *L. lactis* biomass reaction. Thus, we calculated each DNA deoxyribonucleotides molar ratio based on *S. zooepidemicus* ATCC35246 genomic sequence. The calculated molar ratios and the DNA formation reaction were presented in **Table 1**. The results are consistent with the CG content mentioned for this strain (GC% = 41.65).

**S4 Table. Calculation of molar ratio of DNA-forming deoxyribonucleotides.**

| **Deoxyribunocleotide** | **Number in total DNA(bp)** | **Frequency percentage** | **Molar ratio** | **Reference** |
| --- | --- | --- | --- | --- |
| **A** | 631837 | 29.15% | 0.29 | http://microbedb.jp/*Streptococcus equi* subsp. *zooepidemicus* ATCC 35246 |
| **T** | 632859 | 29.20% | 0.29 |  |
| **C** | 449651 | 20.75% | 0.21 |  |
| **G** | 452917 | 20.90% | 0.21 |  |
| **Total** | 21672264 | 100% | 1 |  |
| **DNA formation reaction** | 0.29 datp[c] + 0.21 dctp[c] + 0.21 dgtp[c] + 0.29 dttp[c] -> DNA[c] + ppi[c] | | | |

**Formulation of RNA formation reaction**

In the *L. lactis* biomass reaction, the molar ratio of RNA per gram of biomass was considered as the stoichiometric coefficient. Thus, to formulate the RNA generation reaction, molar ratio of ribonucleotides was calculated. First, the whole-cellular RNA was divided into coding and non-coding types to facilitate the calculations. In order to access the coding and non-coding RNA sequences, the *S. zooepidemicus* ATCC 35246 strain cDNA and non-coding RNA sequences were obtained from the Ensemble Genomes database, respectively. Ribonucleotides molar ratio of coding and non-coding RNA were estimated in **Tables S5** and **S6**. According to the frequency of different types of RNA in prokaryotic cells listed in **Table S7** and each ribonucleotide frequency in coding and non-coding RNAs (**Tables S5** and **S6**), the molar ratio of each ribonucleotide in one mole of whole-cellular RNA was calculated, which is shown in **Table S8**.

**S5 Table. Calculation of molar ratio of non-coding RNA-forming ribonucleotides.**

| **Ribunocleotide** | **Number in coding RNA** | **Frequency percentage** | **Molar ratio** | **Reference** |
| --- | --- | --- | --- | --- |
| **A** | 533317 | 29.04% | 0.29 | http://bacteria.ensembl.org  /Streptococcus_equi_subsp  _zooepidemicus_atcc_35246 |
| **U** | 521132 | 28.38% | 0.28 |  |
| **C** | 356591 | 19.42% | 0.19 |  |
| **G** | 425076 | 23.16% | 0.23 |  |
| **Total** | 1836116 | 100% | 1 |  |

**S6 Table. Calculation of molar ratio of noncoding-RNA-forming ribonucleotides.**

| **Ribunocleotide** | **Number in coding RNA** | **Frequency percentage** | **Molar ratio** | **Reference** |
| --- | --- | --- | --- | --- |
| **A** | 14259 | 24.32% | 0.24 | http://bacteria.ensembl.org  /Streptococcus_equi_subsp  _zooepidemicus_atcc_35246 |
| **U** | 14341 | 24.46% | 0.25 |  |
| **C** | 15158 | 25.86% | 0.26 |  |
| **G** | 14868 | 25.36% | 0.25 |  |
| **Total** | 58626 | 100% | 1 |  |

**S7 Table. Frequency of different types of RNA in the prokaryotic cells.**

| **RNA type** | **mRNA** | **rRNA** | **tRNA** | **Reference** |
| --- | --- | --- | --- | --- |
| **Frequency** | 5% | 80% | 15% | (Neidhardt and Curtiss 1996) |
| **Coding or non-coding** | coding | Non-coding | |  |
| **Frequency** | 5% | 95% | |  |

**S8 Table. Calculation of molar ratio of whole-RNA-forming ribonucleotides.**

| **Ribunocleotide** | **Molar ratio in coding RNA** | **Molar ratio in non-coding RNA** | **Molar ratio in whole-RNA** | **Reference** |
| --- | --- | --- | --- | --- |
| **A** | 0.0145 | 0.228 | 0.2424 | This study |
| **U** | 0.014 | 0.2375 | 0.2514 |  |
| **C** | 0.0095 | 0.247 | 0.2565 |  |
| **G** | 0.015 | 0.2375 | 0.2524 |  |
| **RNA formation reaction** | 0.24atp[c] + 0.26 ctp[c] + 0.25 gtp[c] + 0.25 utp[c] -> RNA-LLA[c] + ppi[c] | | | |

**Formulation of protein formation reaction**

In *L. lactis* biomass reaction, the molar ratio of protein per gram of biomass was considered as the stoichiometric coefficient. Thus, to formulate the protein generation reaction, the molar ratio of amino acids was computated. For this purpose, we obtained *S. zooepidemicus* ATCC 35246 codon preference data from HIVE-Codon Usage Tables (HIVE-CUTs) database. Then the frequency percentage of each codon was calculated based on the number of repetitions of each codon relative to the total codons forming one type of amino acid. The sum of the percentage of frequency of different codons translated into a kind of amino acid gives the percentage of each amino acid frequency. Finally, the percentage of amino acids that make up one protein mole was calculated from the frequency percentage obtained for each amino acid. Details of the calculations are given in **Table S9**. (The data obtained are related to 2119 coding DNA sequences (CDS)).

**S9 Table. Calculation of molar ratio of whole-protein-forming amino acids.**

| **Amino acid** | **Codon sequence** | **Number of repetitions** | **%Frequency of each codon */* total codons of amino acid** | **%Frequency of each amino acid** | **Molar ratio** | **Reference** |
| --- | --- | --- | --- | --- | --- | --- |
| Ala | GCC | 10838 | 1.77% | 8.24% | 0.0824 | This study |
|  | GCA | 13341 | 2.18% |  |  |  |
|  | GCG | 4483 | 0.73% |  |  |  |
|  | GCT | 21769 | 3.56% |  |  |  |
| Arg | CGA | 2646 | 0.43% | 4.25% | 0.0425 |  |
|  | AGG | 3456 | 0.56% |  |  |  |
|  | CGT | 8385 | 1.37% |  |  |  |
|  | CGG | 1576 | 0.26% |  |  |  |
|  | AGA | 5315 | 0.87% |  |  |  |
|  | CGC | 4659 | 0.76% |  |  |  |
| Asn | AAC | 7231 | 1.18% | 4.01% | 0.0401 |  |
|  | AAT | 17303 | 2.83% |  |  |  |
| Asp | GAC | 10982 | 1.80% | 5.64% | 0.0564 |  |
|  | GAT | 23450 | 3.84% |  |  |  |
| Cys | TGC | 1471 | 0.24% | 0.63% | 0.0063 |  |
|  | TCT | 2397 | 0.39% |  |  |  |
| Gln | CAA | 13557 | 2.22% | 4.48% | 0.0448 |  |
|  | CAG | 13811 | 2.26% |  |  |  |
| Glu | GAG | 20105 | 3.29% | 6.40% | 0.064 |  |
|  | GAA | 18985 | 3.11% |  |  |  |
| Gly | GGA | 9353 | 1.53% | 6.43% | 0.0643 |  |
|  | GGT | 13713 | 2.24% |  |  |  |
|  | GGG | 5547 | 0.91% |  |  |  |
|  | GGC | 10700 | 1.75% |  |  |  |
| His | CAG | 3893 | 0.64% | 2.1% | 0.021 |  |
|  | CAT | 8921 | 1.46% |  |  |  |
| Ile | ATT | 26768 | 4.38% | 7.14% | 0.0714 |  |
|  | ATC | 12210 | 2% |  |  |  |
|  | ATA | 4644 | 0.67% |  |  |  |
| Leu | CTT | 13278 | 2.17% | 10.45% | 0.1045 |  |
|  | CTG | 9195 | 1.51% |  |  |  |
|  | TTA | 13844 | 2.26% |  |  |  |
|  | CTA | 9516 | 1.56% |  |  |  |
|  | CTC | 5001 | 0.82% |  |  |  |
|  | TTG | 13004 | 2.13% |  |  |  |
| Lys | AAG | 21800 | 3.57% | 6.75% | 0.0675 |  |
|  | AAA | 19452 | 3.18% |  |  |  |
| Met | ATG | 15016 | 2.46% | 2.46% | 0.0246 |  |
| Phe | TTT | 21601 | 3.53% | 4.29% | 0.0429 |  |
|  | TTC | 4670 | 0.76% |  |  |  |
| Pro | CCG | 2563 | 0.42% | 3.38% | 0.0338 |  |
|  | CCC | 2207 | 0.36% |  |  |  |
|  | CCA | 8076 | 1.32% |  |  |  |
|  | CCT | 7821 | 1.28% |  |  |  |
| Ser | AGT | 6925 | 1.13% | 6.30% | 0.063 |  |
|  | TCA | 9859 | 1.61% |  |  |  |
|  | AGC | 7917 | 1.30% |  |  |  |
|  | TCG | 2012 | 0.33% |  |  |  |
|  | TCC | 3403 | 0.56% |  |  |  |
|  | TCT | 8400 | 1.37% |  |  |  |
| STOP | TAG | 579 | 0.09% | 0.34% | 0.0034 |  |
|  | TGA | 355 | 0.06% |  |  |  |
|  | TAA | 1166 | 0.19% |  |  |  |
| Thr | ACA | 11734 | 1.92% | 5.55% | 0.0555 |  |
|  | ACT | 8284 | 1.35% |  |  |  |
|  | ACC | 9910 | 1.62% |  |  |  |
|  | ACG | 4045 | 0.66% |  |  |  |
| Trp | TGG | 5167 | 0.84% | 0.84% | 0.0084 |  |
| Tyr | TAT | 15979 | 2.61% | 3.77% | 0.0377 |  |
|  | TAC | 7090 | 1.16% |  |  |  |
| Val | GTC | 8361 | 1.37% | 6.55% | 0.0655 |  |
|  | GTG | 9054 | 1.48% |  |  |  |
|  | GTT | 16665 | 2.73% |  |  |  |
|  | GTA | 5917 | 0.97% |  |  |  |
| Total | 611366 |  | | 100% | 1 |  |
| Protein formation reaction | 0.0824 alatrna[c] + 0.0425 argtrna[c] + 0.0401 asntrna[c] + 0.06 asptrna[c] + 0.0063 cystrna[c] + 0.448 glntrna[c] + 0.064 glutrna[c] + 0.0643 glytrna[c] + 2.0 gtp[c] + 2 h2o[c] + 0.0215 histrna[c] + 0.0714 iletrna[c] + 0.105 leutrna[c] + 0.068 lystrna[c] + 0.026 mettrna[c] + 0.043 phetrna[c] + 0.034 protrna[c] + 0.063 sertrna[c] + 0.056 thrtrna[c] + 0.084 trptrna[c] + 0.038 tyrtrna[c] + 0.066 valtrna[c] -> 2.0 gdp[c] + 2.0 h[c] + 2.0 pi[c] + prot-LLA[c] + 0.0824 trnaala[c] + 0.0425 trnaarg[c] + 0.0401 trnaasn[c] + 0.06 trnaasp[c] + 0.0063 trnacys[c] + 0.0643 trnagly[c] + 0.0215 trnahis[c] + 0.0714 trnaile[c] + 0.105 trnaleu[c] + 0.068 trnalys[c] + 0.026 trnamet[c] + 0.043 trnaphe[c] + 0.034 trnapro[c] + 0.063 trnaser[c] + 0.056 trnathr[c] + 0.084 trnatrp[c] + 0.038 trnatyr[c] + 0.066 trnaval[c] + 0.512 trnaglu[c] | | | | | |

**The estimation of non-growth associated maintenance parameter**

The amount of energy required for cell survival and maintenance is known as non-growth associated maintenance (NGAM), which is one of the essential energetic parameters for genome-scale metabolic model reconstruction. According to the Protocol of Genome-Scale Metabolic Model Reconstruction (Thiele and Palsson, 2010), NGAM is equal to the y-intercept of line fitting the data points when the plot was drawn based on ATP consumption rate(mmol.gCDW^-1^.h^-1^) on growth rate(h^-1^). The available data for the target strain in chemostat culture were based on glucose consumption rate (Hamilton et al., 1979) (Blank et al., 2005). Thus, at first a diagram of glucose consumption rate on cell growth rate was plotted and the y-intercept of line fitting was calculated, which was equal to 0.68 mmol.gCDW^-1^.h^-1^ **(Fig S1)**. Then, the ATP yield relative to glucose (the amount of ATP produced per mole of glucose) was calculated using the i*ZN522* model equaling to 20 mmol_ATP_.mmol_glucose_^-1^. Finally, the y-intercept obtained from the graph was converted to NGAM using ATP yield. We adjusted NGAM at 13.6 mmol.grCDW^-1^.h^-1^ to simulate *S. zooepidemicus* growth on defined condition.


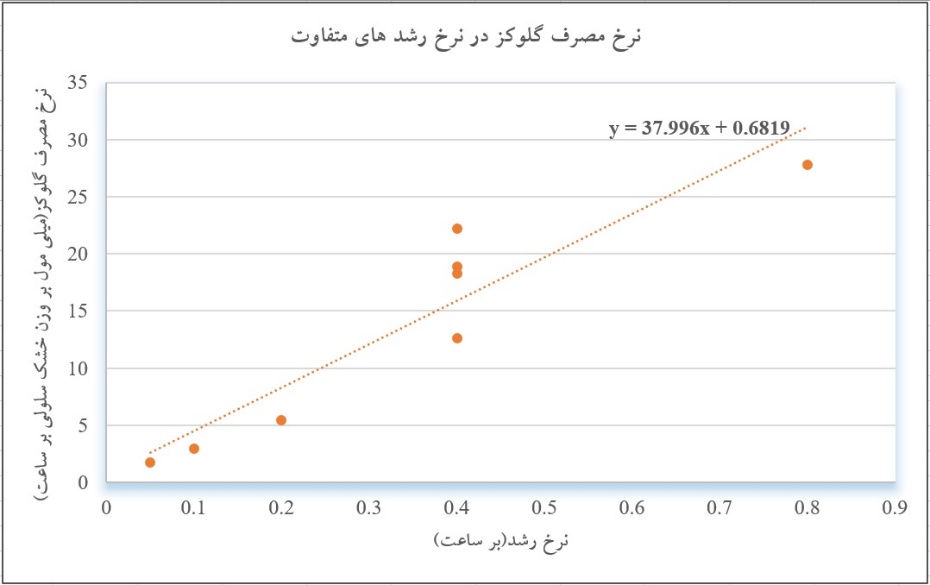


**Glucose consumption rates at various growth rates**

Glucose consumption rate (mmol.g_CDW_^-1^.h^-1^)

Growth rate (h^-1^)

**S1 Fig. Relationship between glucose consumption rate (mmol·gCDW⁻¹·h⁻¹) and cell growth rate (h⁻¹) in chemostat culture.** The fitted linear regression (y = 37.996x + 0.6819) estimates NGAM as the y-intercept, equal to 0.6819 mmol·gCDW⁻¹·h⁻¹.
